# The Validity of Quadriceps Muscle Thickness as a Nutritional Risk Indicator in Patients with Stroke

**DOI:** 10.3390/nu16040540

**Published:** 2024-02-15

**Authors:** Motoki Maruyama, Yuki Kagaya, Sota Kajiwara, Takuto Oikawa, Manabu Horikawa, Mika Fujimoto, Masahiro Sasaki

**Affiliations:** 1 Department of Rehabilitation, Akita Cerebrospinal and Cardiovascular Center, 6-10 Senshu-Kubota-Machi, Akita 010-0874, Japan; 2 Department of Physical Therapy, Akita University Graduate School of Health Science, 1-1-1 Hondo, Akita 010-8543, Japan; 3 Department of Rehabilitation Medicine, Akita Cerebrospinal and Cardiovascular Center, 6-10 Senshu-Kubota-Machi, Akita 010-0874, Japan; 4 Department of Nutrition, Akita Cerebrospinal and Cardiovascular Center, 6-10 Senshu-Kubota-Machi, Akita 010-0874, Japan; fujimoto-mika@akita-hos.or.jp

**Keywords:** nutritional risk, muscle thickness, stroke, rehabilitation, activities of daily living

## Abstract

This study aimed to investigate whether quadriceps muscle thickness (QMT) is useful for nutritional assessment in patients with stroke. This was a retrospective cohort study. Nutritional risk was assessed using the Geriatric Nutritional Risk Index (GNRI), with GNRI < 92 indicating a risk of malnutrition and GNRI ≥ 92 indicating normal conditions. Muscle mass was assessed using QMT and calf circumference (CC). The outcome was Functional Independence Measure (FIM) effectiveness. The cutoff values of QMT and CC for discriminating between high and low GNRI were determined using the receiver operating characteristic curve. The accuracy of the nutritional risk discrimination model was evaluated using the Matthews correlation coefficient (MCC). Multiple regression analysis was performed to assess the relationship between nutritional risk, as defined by QMT and CC, and FIM effectiveness. A total of 113 patients were included in the analysis. The cutoff values of QMT and CC for determining nutritional risk were 49.630 mm and 32.0 cm for men (MCC: 0.576; 0.553) and 41.185 mm and 31.0 cm for women (MCC: 0.611; 0.530). Multiple regression analysis showed that only nutritional risk defined by QMT was associated with FIM effectiveness. These findings indicate that QMT is valid for assessing nutritional risk in patients with stroke.

## 1. Introduction

Ultrasound (US) has become widely used in clinical practice due to its ability to assess the quantity and quality of skeletal muscles [1]. Patients with stroke are prone to secondary skeletal muscle degeneration; therefore, US is used in clinical practice for skeletal muscle assessment. In addition, it is expected that the measurement of muscle thickness (MT) with US will be applied in nutritional assessment, which is essential in rehabilitation nutrition [2]. The Global Leadership Initiative on Malnutrition, which serves as diagnostic criteria for malnutrition, includes reduced muscle mass in its diagnostic criteria [3]. Since MT correlates with muscle mass [1], there is the potential for assessing the risk of malnutrition conveniently using US. However, no studies have used MT in the nutritional assessment of patients with stroke, and its usefulness is unknown.

The prevalence of malnutrition in patients with stroke ranges from 6.1% to 62% [4]. Malnutrition has been reported to have a negative effect on mortality, physical function, home discharge rates, and cognitive function [5,6,7,8,9,10,11,12,13,14,15,16,17,18,19]. Additionally, the concept of a malnutrition–disability cycle has been proposed in a recent review, which suggests a negative interaction between malnutrition and physical disability [20]. Therefore, assessing nutritional status is essential for predicting functional prognosis in patients with stroke.

The Geriatric Nutritional Risk Index (GNRI) and Controlling Nutritional Status (CONUT) have been widely used in clinical practice as measures of nutritional status [21,22]. Malnutrition and nutritional risk determined by the CONUT and GNRI have been reported to be associated with poor outcomes in patients with stroke [5,6,7,8,9,10,11,12,13,14]. However, CONUT and GNRI require laboratory data, which poses a challenge in terms of simplicity. In clinical practice, skeletal muscle mass is used as a measure in nutritional assessment. Nutritional assessment using calf circumference (CC) has been used as a non-invasive method [23,24,25]. Although CC is a simple assessment, it may not accurately assess skeletal muscle mass in individuals with edema or obesity [26,27]. In contrast, skeletal muscle assessment using US has recently gained attention as a simple and non-invasive nutritional assessment tool that is less susceptible to the effects of edema and subcutaneous fat. MT measured with US has been reported to have potential applications in nutritional assessment [2]. Therefore, MT measured with US may be a valuable tool in nutritional assessment. However, it remains unclear as to whether nutritional assessment using MT is useful in patients with stroke who are prone to skeletal muscle atrophy [28,29]. In addition, the association between nutritional risk determined using MT and outcomes in convalescent rehabilitation wards has not been examined; hence, its predictive validity is unknown. We believe that examining the concurrent and predictive validity of MT in the nutritional assessment of patients with stroke will provide insights into stroke rehabilitation.

Therefore, this study aimed to determine whether quadriceps MT (QMT) on the non-paretic side was more useful than CC, which has conventionally been used to indicate nutritional risk in patients with stroke admitted to convalescent rehabilitation wards. In addition, we examined the association between nutritional risk—determined with QMT and CC on the non-paretic side—and the rate of improvement in physical function in a convalescent rehabilitation ward.

## 2. Materials and Methods

### 2.1. Study Design and Participants

This was a retrospective cohort study. A total of 221 patients with stroke who were admitted to a convalescent rehabilitation ward between July 2021 and November 2022 were enrolled in this study. The exclusion criteria included age < 65 years, a history of stroke, recent surgical procedures, severe inflammation (C-reactive protein ≥ 3.0 mg/dL [30]), the presence of other neurological diseases, previous lower limb surgery, and missing data.

### 2.2. Data Collection

Participants’ characteristics, including age, sex, body mass index, stroke subtype, number of days from onset, motor paralysis, and activities of daily living (ADLs), were collected from electronic records. Motor paralysis of the lower limb was assessed using the Brunnstrom recovery stage (BRS) [31]. ADL was assessed using the Functional Independence Measure [32]. All data were collected within one week of admission to the convalescent rehabilitation ward.

### 2.3. Nutritional Risk Assessment

Nutritional risk was assessed using the GNRI [21] upon admission to the convalescent rehabilitation ward. The GNRI was calculated using serum albumin levels, ideal body weight, and actual body weight based on the following formula: 14.89 × serum albumin level (g/dL) + 41.7 × actual body weight (kg)/ideal body weight (kg) [21]. In a previous study, a high correlation was observed between GNRI calculated using the Lorenz formula for ideal body weight and that calculated with a BMI of 22 [33]. Therefore, the ideal body weight was calculated as height (m)^2^ × 22 [33]. If the actual body weight exceeded the ideal body weight, the actual-body-weight-to-ideal-body-weight ratio was set as 1 [21]. A GNRI less than 92 was defined as a risk of malnutrition, and a GNRI more than 92 was defined as normal [34].

### 2.4. Muscle Mass Assessment

MT was assessed using US (Noblus, Hitachi, Ltd., Tokyo, Japan). The instrument settings were configured with a 20 dB gain, 70 Hz dynamic range, 9.0 MHz frequency, and 7.0 cm depth for all subjects. The focus was set on the deep fascia of each muscle or bone surface for each subject. Measurements were performed with the subjects in the supine position, with the hip joint in the middle position, and the knee joint extended while applying a water-soluble gel to the skin surface of the thigh. The probe was placed in light contact perpendicular to the skin to avoid muscle deformation. The target muscles were the quadriceps femoris on the non-paretic side, which is the most frequently studied muscle group according to a systematic review [35]. The rectus femoris, vastus intermedius, and vastus lateralis were measured at the midpoint between the greater trochanter and lateral condyle, whereas the vastus medialis was measured at 30% lower [36]. MT was defined as the linear distance from the superficial fascia to the deep fascia or bone surface. QMT was calculated as the sum of the rectus femoris, vastus intermedius, vastus lateralis, and vastus medialis [36]. Additionally, the thickness of the subcutaneous fat of the anterior thigh on the non-paretic side was measured at the same time. Measurements of muscle and subcutaneous fat thickness were each taken three times, and the average value was calculated. QMT was measured by a single examiner and showed a high intra-rater correlation coefficient (ICC = 0.99).

The maximum CC on the non-paretic side was measured at the same time. CC on the non-paretic side has been used as a nutritional screening tool for patients with stroke [23]. CC was measured to the nearest 0.5 cm with participants in the supine position. QMT and CC were assessed within one week of admission to the convalescent rehabilitation ward.

### 2.5. Outcome

The outcome was the rate of improvement in ADLs. ADLs were assessed using FIM, which is widely used to assess ADLs in patients with stroke [32]. FIM consists of 13 motor and 5 cognitive domains (FIM-M and FIM-C, respectively). Each item is rated on a scale of 1–7 points. FIM-M and FIM-C are rated on scales of 13–91 and 5–35 points, respectively. FIM effectiveness was calculated from FIM-M scores at admission and discharge [37] using the following formula: (FIM-M at discharge − FIM-M at admission)/(91 − FIM-M at admission) × 100 [37]. FIM effectiveness is an indicator of improvement in ADLs and is used as an outcome measure in patients undergoing rehabilitation. FIM effectiveness considers the FIM score at the time of admission and does not show a ceiling effect compared with FIM gain. FIM scores upon admission and discharge were assessed by nurses accustomed to scoring FIM. FIM was assessed within one week after admission to, as well as within one week before discharge from, the convalescent rehabilitation ward.

### 2.6. Rehabilitation Program and Nutritional Management

The rehabilitation program was implemented for up to 3 h per day, depending on the patients’ physical function. The program included physical, occupational, and speech and language therapy, as required. For example, physical therapy included a joint range of motion exercise, resistance training, aerobic exercise, and ADL training.

Nutritional management during hospitalization was provided by a dedicated registered dietitian at the ward according to the physical condition of the patients. Those with malnutrition were offered a high-energy, high-protein diet, whereas those with obesity were offered a high-protein, energy-restricted diet to maintain muscle mass.

### 2.7. Sample Size Calculation

The sample size was calculated using G*Power 3.1. Considering an effect size of 0.3, an alpha error of 0.05, and a power of 0.80, the number of samples required to reject the null hypothesis was 82.

### 2.8. Statistical Analysis

Parametric variables were presented as means and standard deviations, whereas non-parametric variables were presented as medians and interquartile ranges. The comparison of patient characteristics between men and women was examined using *t*-test, Mann–Whitney U test, and Fisher’s exact test. The correlation between QMT and CC on the non-paretic side and GNRI was examined using Pearson’s correlation coefficient. The cutoff values of QMT and CC on the non-paretic side for discriminating between the risk of malnutrition and normal conditions were determined using the receiver operating characteristic curve. The cutoff values were calculated using Youden’s index. Additionally, the accuracy of the model for nutritional risk discrimination using QMT and CC on the non-paretic side was evaluated with the Matthews correlation coefficient (MCC) [38]. MCC measures the model’s predictive ability and takes values from −1 to 1. The higher the model’s predictive accuracy is, the closer it is to 1 [38]. The calculated cutoff values were used to categorize participants into two groups: those at nutritional risk and those considered normal. A comparison of FIM effectiveness between patients at nutritional risk and normal patients, determined by the calculated cutoff values for QMT and CC, was investigated using the Mann–Whitney U test. The correlation between FIM effectiveness and each variable was examined using Spearman’s rank correlation analysis. Multiple regression analysis was performed to examine the association between nutritional risk—defined by QMT and CC on the non-paretic side (nutritional risk-QMT and nutritional risk-CC, respectively)—and FIM effectiveness in the convalescent rehabilitation ward. FIM effectiveness was the dependent variable. The independent variables included nutritional risk-QMT and nutritional risk-CC. The covariates included age, sex, BRS (lower limb), and days from onset. Multicollinearity was assessed using the variance inflation factor (VIF), with VIF < 3.0 indicating no multicollinearity. Statistical analysis was performed using IBM SPSS version 28 (IBM, Armonk, NY, USA). The significance level was set at 0.05.

### 2.9. Ethics

This study was approved by the Ethics Committee of Akita Cerebrospinal and Cardiovascular Center (approval number 23–30). All procedures were performed in accordance with the principles and guidelines of the Declaration of Helsinki. The subjects were informed that they could withdraw from this study at any time. Informed consent was obtained from all subjects involved in the study.

## 3. Results

**Figure 1:**
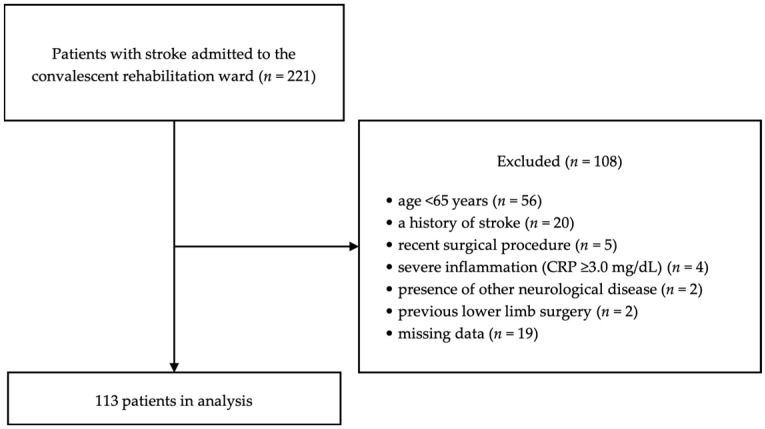
Flowchart of participant exclusion.

Of the 221 patients with stroke who were admitted to the convalescent rehabilitation ward, 108 were excluded due to being aged under 65 years (*n* = 56), having a history of stroke (*n* = 20), recent surgical procedures (*n* = 5), severe inflammation (*n* = 4), the presence of other neurological diseases (*n* = 2), previous lower limb surgery (*n* = 2), and missing data (*n* = 19). Thus, 113 patients were included in the analysis (Figure 1).

Table 1 shows the basic information for each sex. The median age was 76.0 (71.0–82.0) years. The stroke subtypes were cerebral infarction in 75 patients, cerebral hemorrhage in 33 patients, and subarachnoid hemorrhage in 5 patients. The GNRI score was 94.3 ± 7.0. The percentage of patients with the risk of malnutrition was 35.4% (*n* = 40). Significant differences were found in QMT and subcutaneous fat thickness between men and women (QMT: *p* = 0.028; subcutaneous fat thickness: *p* < 0.001). QMT and CC on the non-paretic side were 53.6 ± 11.8 mm and 32.7 ± 2.9 cm for men and 48.6 ± 12.0 mm and 32.2 ± 3.5 cm for women, respectively. The median subcutaneous fat thickness on the non-paretic side was 3.51 (2.71–4.61) and 6.45 (5.17–7.96) mm for men and women, respectively.

Table 2 shows the correlation between QMT and CC on the non-paretic side and GNRI. A significant association was observed between QMT on the non-paretic side and GNRI for each sex (men: *r* = 0.583, *p* < 0.001; women: *r* = 0.650, *p* < 0.001). Additionally, a significant association was observed between CC on the non-paretic side and GNRI for each sex (men: *r* = 0.580, *p* < 0.001; women: *r* = 0.601, *p* < 0.001).

**Figure 2:**
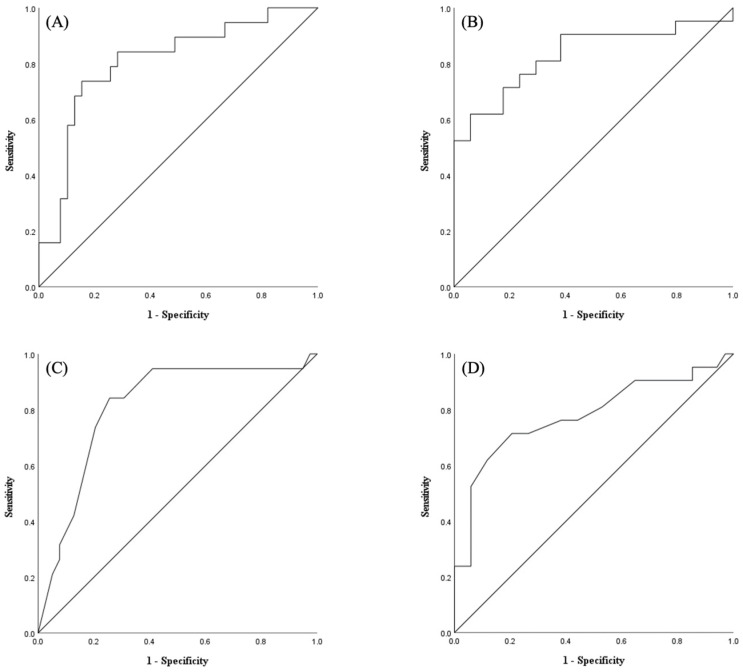
The optimal cutoff values of QMT and CC for nutritional risk were as follows: (**A**) 49.630 mm in men (sensitivity: 0.737; specificity: 0.846; area under the curve (AUC): 0.807; Matthews correlation coefficient (MCC): 0.576) and (**B**) 41.185 mm in women (sensitivity: 0.619; specificity: 0.941; AUC: 0.831; MCC: 0.611). The optimal cutoff values of CC for nutritional risk were as follows: (**C**) 32.0 cm in men (sensitivity: 0.842; specificity: 0.744; AUC: 0.811; MCC: 0.553) and (**D**) 31.0 cm in women (sensitivity: 0.714; specificity: 0.794; AUC: 0.779; MCC: 0.530).

Figure 2 shows the receiver operating characteristic curve of QMT and CC on the non-paretic side for determining high and low GNRI. The cutoff values of QMT on the non-paretic side for discriminating between high and low GNRI were 49.630 mm for men (sensitivity: 0.737; specificity: 0.846; area under the curve (AUC): 0.807) and 41.185 mm for women (sensitivity: 0.619; specificity: 0.941; AUC: 0.831). Additionally, the cutoff values of CC on the non-paretic side were 32.0 cm for men (sensitivity: 0.842; specificity: 0.744; AUC: 0.811) and 31.0 cm for women (sensitivity: 0.714; specificity: 0.794; AUC: 0.779). The MCCs of QMT and CC on the non-paretic side were 0.576 and 0.553 for men and 0.611 and 0.530 for women, respectively.

Based on the univariate analysis, FIM effectiveness was significantly lower in the group defined as nutritional risk based on the cutoff values for QMT and CC compared to the normal group (QMT: *p* < 0.001; CC: *p* = 0.016). Additionally, FIM effectiveness showed a significant correlation with BRS (rho = 0.200; *p* = 0.034) and days from onset (rho = −0.318; *p* < 0.001). Table 3 and Table 4 show the relationship between nutritional risk, as defined by QMT and CC on the non-paretic side, and FIM effectiveness. All variables exhibited VIF < 3.0, and there was no multicollinearity among the independent variables. Multiple regression analysis showed that BRS and nutritional risk-QMT were significantly associated with FIM effectiveness (BRS: β = 0.24; B = 4.98; 95% confidence interval (CI): 1.18–8.78; *p* = 0.011. Nutritional risk-QMT: β = −0.25; B = −14.81; 95% CI: −26.07 to −3.55; *p* = 0.010). In contrast, nutritional risk-CC was not significantly associated with FIM effectiveness (β = −0.12; B = −6.84; 95% CI: −17.16 to 3.49; *p* = 0.192).

## 4. Discussion

In this study, the utility of QMT measured with US as an indicator of nutritional risk was compared with the conventionally used CC in patients with stroke admitted to a convalescent rehabilitation ward. In addition, to examine the relationship between nutritional risk, as defined by QMT on the non-paretic side, and the rate of improvement in ADLs, multiple regression analysis was performed. The results showed three novel findings regarding QMT in patients with stroke. QMT on the non-paretic side was correlated with GNRI, and cutoff values for discriminating nutritional risk were calculated for each sex. The discriminability was similar between QMT and CC in men and slightly better for QMT than CC in women. Nutritional risk, as determined with QMT on the non-paretic side, was negatively associated with the rate of improvement in ADLs in the convalescent rehabilitation ward.

In this study, the prevalence of the risk of malnutrition was 35.4% in patients with stroke admitted to the convalescent rehabilitation ward. In a previous study, the prevalence of the risk of malnutrition determined using GNRI was 40.5% [9], consistent with the findings of our study. Patients with stroke are prone to malnutrition as a result of inadequate energy intake and inflammation early in stroke onset. In this study, moderate-to-severe nutritional risk was found in one of every three patients. These results demonstrate the importance of nutritional assessment in patients with stroke.

A significant positive correlation was observed between QMT on the non-paretic side and GNRI. Previous studies have reported a correlation between MT measured with US in various muscle groups, such as the masseter, temporalis, lingual muscles, quadriceps, and tibialis anterior muscles, and malnutrition or nutritional risk [39,40,41,42,43]. These studies were conducted in intensive care units, patients hospitalized for hip fractures, and older subjects [39,40,41,42,43]. These findings are consistent with those of this study, which showed that QMT on the non-paretic side was correlated with nutritional risk in patients with stroke. Muscle atrophy has been reported to occur on both the paretic and non-paretic sides of the lower limb in patients with stroke from the early stages [28,29]. Therefore, this study indicates that QMT measured with US may serve as a surrogate marker for assessing nutritional risk, even in patients with stroke who are susceptible to muscle atrophy. In this study, the optimal cutoff values for QMT on the non-paretic side to discriminate nutritional risk were 49.630 mm for men and 41.185 mm for women. Specificity was higher than sensitivity for both men and women, indicating a lower rate of false positives. These optimal cutoff values were determined upon admission to the convalescent rehabilitation ward. Further studies are needed to explore whether these values are applicable at different stages of patient care.

In women, the discriminability of nutritional risk was slightly higher in QMT than in CC. This difference may be influenced by subcutaneous fat and edema. Herein, subcutaneous fat thickness was higher in women than in men. No significant correlation was observed between CC and gastrocnemius MT measured with US in older women with excess weight [27]. In addition, CC in older adults has been reported to be affected by edema [26], which is associated with older age and female sex [44]. The increased thickness of subcutaneous fat due to obesity and edema may lead to an overestimation of CC. By contrast, US can separately measure muscles and subcutaneous fat, and it is less affected by edema. Therefore, it is possible that QMT more accurately reflects skeletal muscle mass in women, and the discriminability of nutritional risk was higher in QMT than in CC.

Nutritional risk, as determined using QMT on the non-paretic side (men: ≤49.630 mm; women: ≤41.185 mm), was significantly associated with the rate of improvement in ADLs. Previous studies have shown that MT measured using US correlated with muscle strength and walking independence [45,46,47,48], and muscle mass was associated with ADLs [49]. Considering our results, QMT may reflect nutritional risk in addition to muscle strength and walking independence. Therefore, the nutritional risk defined by QMT may be associated with the rate of improvement in ADLs. In this study, nutritional risk, as determined using CC on the non-paretic side, was not significantly associated with the rate of improvement in ADLs. Therefore, it is possible that QMT on the non-paretic side is a nutritional indicator with better concurrent and predictive validity than CC on the non-paretic side in patients with stroke.

MT measured using US has shown a high correlation with skeletal muscle mass measured using magnetic resonance imaging or computed tomography [1,50]. US has several advantages, including being a low-cost, low-risk, non-invasive, and portable tool [51]. Additionally, it can be used by allied health professionals, such as physical therapists and registered dietitians. This study’s results suggest that MT measured with US can be used as a simple, non-invasive surrogate marker of nutritional risk in patients with stroke.

This study has several limitations. First, it was a single-center study, and due to the small sample size, separate analyses by sex could not be conducted in multivariate analysis. Second, this was a retrospective cohort study conducted in a convalescent rehabilitation ward. Therefore, the generalizability of these results to different facilities and disease stages may be limited. Additionally, the measurement of MT requires US, further limiting the generalizability. Further studies are needed to examine whether these findings can be applied to different facilities and phases with a large sample size.

## 5. Conclusions

This study examined the utility of QMT on the non-paretic side in assessing nutritional risk in patients with stroke. In addition, we defined a cutoff value to determine nutritional risk and investigated the association between nutritional risk, defined by QMT, and the rate of improvement in ADLs. QMT was associated with GNRI in both men and women, and the discriminability was slightly higher than that of CC in women. Furthermore, nutritional risk, as defined by QMT, was associated with the rate of recovery of physical function. These findings suggest that QMT on the non-paretic side could be a surrogate marker for nutritional risk and has predictive validity for the recovery rate of ADLs in patients with stroke.

## Figures and Tables

**Table 1:** Patient characteristics for each sex.

Variables	Total (*N* = 113)	Men (*N* = 58)	Women (*N* = 55)	*p*
Age, years (IQR)	76.0 (71.0–82.0)	74.0 (71.0–80.8)	78.0 (72.0–83.0)	0.456 ^a^
Body mass index, kg/m^2^ (SD)	22.6 (3.6)	22.3 (3.4)	22.8 (3.8)	0.404 ^b^
Stroke subtype, *n* (%)				0.197 ^c^
Cerebral infarction	75 (66.4)	37 (63.8)	38 (69.1)	
Cerebral hemorrhage	33 (29.2)	20 (34.5)	13 (23.6)	
Subarachnoid hemorrhage	5 (4.4)	1 (1.7)	4 (7.3)	
Brunnstrom recovery stage (lower limb) (IQR)	5 (4–6)	5 (5–6)	5 (4–6)	0.220 ^a^
Days from onset, days (IQR)	17 (12–22)	17 (12–22)	16 (13–23)	0.854 ^a^
Laboratory data				
Serum albumin, g/dL (SD)	3.7 (0.4)	3.7 (0.4)	3.6 (0.4)	0.321 ^b^
C-reactive protein, mg/dL (IQR)	0.13 (0.06–0.61)	0.11 (0.06–0.34)	0.17 (0.07–0.74)	0.186 ^a^
FIM-total upon admission, score (IQR)	73 (45–92)	75 (46–99)	69 (47–90)	0.352 ^a^
FIM-motor upon admission, score (IQR)	50.0 (32.0–69.0)	53.5 (33.3–69.8)	46.0 (30.5–64.5)	0.267 ^a^
FIM-motor upon discharge, score (IQR)	76.0 (55.0–84.0)	77.0 (55.8–84.0)	75.0 (52.0–84.5)	0.579 ^a^
FIM effectiveness, %	54.5 (34.6–73.3)	51.1 (32.0–71.1)	57.1 (36.4–73.8)	0.636 ^a^
GNRI, score (SD)	94.3 (7.0)	94.8 (6.8)	93.7 (7.3)	0.414 ^b^
Risk of malnutrition (<92), *n* (%)	40 (35.4)	19 (32.8)	21 (38.2)	
Normal (≥92), *n* (%)	73 (64.6)	39 (67.2)	34 (61.8)	
QMT on the non-paretic side, mm (SD)	51.2 (12.1)	53.6 (11.8)	48.6 (12.0)	0.028 ^b^
CC on the non-paretic side, cm (SD)	32.5 (3.2)	32.7 (2.9)	32.2 (3.5)	0.392 ^b^
SFT on the non-paretic side, mm (IQR)	4.71 (3.25–6.65)	3.51 (2.71–4.61)	6.45 (5.17–7.96)	<0.001 ^a^

SD, standard deviation; IQR, interquartile range; FIM, Functional Independence Measure; GNRI, Geriatric Nutritional Risk Index; QMT, quadriceps muscle thickness; CC, calf circumference; SFT, subcutaneous fat thickness. a: Mann–Whitney U test; b: *t*-test; c: Fisher’s exact test.

**Table 2:** Correlation between QMT and CC on non-paretic side and GNRI.

Variables	Men (*N* = 58)		Women (*N* = 55)	
	Correlation Coefficient (95% CI)	*p*	Correlation Coefficient (95% CI)	*p*
QMT on the non-paretic side	0.583 (0.382, 0.731)	<0.001	0.650 (0.465, 0.781)	<0.001
CC on the non-paretic side	0.580 (0.379, 0.729)	<0.001	0.601 (0.399, 0.747)	<0.001

QMT, quadriceps muscle thickness; CC, calf circumference; GNRI, Geriatric Nutritional Risk Index; CI, confidence interval.

**Table 3:** Relationship between nutritional risk defined by QMT and FIM effectiveness.

Variables	β	B	95% CI	*p*	VIF
Age	−0.13	−0.50	−1.23, 0.22	0.172	1.17
Sex (women)	0.05	2.90	−6.74, 12.54	0.552	1.04
BRS (lower limb)	0.24	4.98	1.18, 8.78	0.011	1.11
Days from onset	−0.18	−0.55	−1.13, 0.03	0.061	1.15
Nutritional risk-QMT	−0.25	−14.81	−26.07, −3.55	0.010	1.21

β, standardized partial regression coefficient; B, partial regression coefficient; CI, confidence interval; VIF, variance inflation factor; FIM; Functional Independence Measure; BRS, Brunnstrom recovery stage; QMT, quadriceps muscle thickness. Covariates: age, sex (women), BRS (lower limb), days from onset. Adjusted R^2^: 0.166; Durbin–Watson: 1.88; normality of residuals: *p* = 0.67.

**Table 4:** Relationship between nutritional risk defined by CC and FIM effectiveness.

Variables	β	B	95% CI	*p*	VIF
Age	−0.16	−0.63	−1.38, 0.11	0.094	1.17
Sex (women)	0.07	3.85	−5.99, 13.68	0.440	1.04
BRS (lower limb)	0.22	4.68	0.74, 8.62	0.020	1.14
Days from onset	−0.24	−0.75	−1.31, −0.19	0.009	1.04
Nutritional risk-CC	−0.12	−6.84	−17.16, 3.49	0.192	1.12

β, standardized partial regression coefficient; B, partial regression coefficient; CI, confidence interval; VIF, variance inflation factor; FIM; Functional Independence Measure; BRS, Brunnstrom recovery stage; CC, calf circumference. Covariates: age, sex (women), BRS (lower limb), days from onset. Adjusted R^2^: 0.127; Durbin–Watson: 1.81; normality of residuals: *p* = 0.56.

## Data Availability

The data are not publicly available due to opt-out restrictions. Data are contained within the article.
